# Factors associated with breastfeeding in infants with trisomy 21

**DOI:** 10.1590/2317-1782/e20240267en

**Published:** 2025-12-01

**Authors:** Larissa Melgaço Campos, Ana Elisa Ribeiro Fernandes, Andréa Rodrigues Motta, Renata Maria Moreira Moraes Furlan

**Affiliations:** 1 Programa de Pós-graduação em Ciências Fonoaudiológicas, Universidade Federal de Minas Gerais – UFMG - Belo Horizonte (MG), Brasil.; 2 Departamento de Pediatria, Faculdade de Medicina, Universidade Federal de Minas Gerais – UFMG - Belo Horizonte (MG), Brasil.; 3 Departamento de Fonoaudiologia, Universidade Federal de Minas Gerais – UFMG - Belo Horizonte (MG), Brasil.

**Keywords:** Breast Feeding, Down Syndrome, Stomatognathic System, Speech, Language and Hearing Sciences, Tongue

## Abstract

**Purpose:**

To investigate the duration of breastfeeding (BF) and exclusive BF in infants with T21 and to verify the association between clinical and myofunctional orofacial conditions and the presence of BF and exclusive BF in the sixth month and in the first year of life.

**Methods:**

This was a prospective, longitudinal, observational study with 90 infants with T21. Parents answered two questionnaires about clinical and BF conditions, one in the sixth and the other in the 12th month of the infant's life. Habitual lip and tongue posture was obtained through videos, and clinical history was obtained through medical records. The presence of BF and exclusive BF in the sixth and 12th months was associated with clinical and myofunctional orofacial conditions using Pearson's chi-square test, with a 5% significance level.

**Results:**

There were associations between male infants and the presence of BF/exclusive BF at the sixth month; formula use and absence of BF; BF in the first hour and the presence of BF in the first year; lung disease and BF in the first year; BF difficulties and BF in the sixth month; BF in the sixth month and latching difficulties; low milk production and absence of BF in the first year; pacifier use and absence of BF in the sixth month and of BF in the first year.

**Conclusion:**

Sex, lung disease, formula use, BF in the first hour of life, self-reported BF difficulties, low milk production, and pacifier use were factors associated with BF in infants with T21.

## INTRODUCTION

Trisomy 21 (T21), or Down syndrome, is a genetic condition caused by the triplication of chromosome 21. One of its clinical manifestations is muscle hypotonia^(1)^, which includes orofacial structures such as lips and tongue. This hypotonia alters the usual posture of these structures and impairs the stomatognathic functions of sucking, chewing, and swallowing, which can lead to difficulties in breastfeeding (BF) and the later introduction of solid foods^(2)^. Furthermore, babies with T21 may have associated comorbidities that can also influence these processes, such as congenital heart disease^(3)^, with increased metabolic demand and tachydyspnea during sucking, alterations in the digestive and respiratory systems, among other changes^(4)^.

Infants with T21 may have difficulty breastfeeding due to muscle hypotonia^(5)^. The literature reports shorter BF in infants with T21^(6,7)^, but few studies have addressed the causes of BF difficulties and early weaning, which may be associated with the specificities of the syndrome and the various associated comorbidities. Furthermore, there is a lack of preparation among health professionals to deal with BF in atypical situations, which may discontinue BF due to a lack of information^(8)^.

Studies indicate that infants with T21 breastfeed less frequently than the typical populations due to clinical conditions that hinder the BF process^(9)^. An Italian study^(6)^ revealed that the average BF duration among children with T21 was 54 days, compared to 164 days in the control group. Another study, conducted in Chile, found a higher frequency of exclusive BF until the sixth month (46.6%), with a 71.2% prevalence of BF, and 96% of mothers breastfed for at least 1 month^(10)^. In contrast, a study^(11)^ in Puerto Rico found that 84.6% of mothers of infants with T21 chose not to breastfeed or discontinued BF because the infants had difficulty sucking and the mothers did not receive BF guidance during hospitalization.

The World Health Organization (WHO) recommends exclusive BF up to 6 months old because breast milk meets all the nutritional needs of the baby at this age^(12)^. Furthermore, BF contributes to the development of orofacial myofunctional structures^(13)^, which, in the case of T21, are hypotonic, making BF even more beneficial^(13)^. Therefore, greater knowledge and preparation of health professionals is necessary to guide these babies’ mothers regarding BF, since this initial stage, if not successfully completed, can affect later stages, such as the acceptance of the first foods offered^(14)^.

Hence, this study aimed to investigate the duration of BF and exclusive BF in infants with T21 and to verify the association between orofacial clinical and myofunctional conditions with BF and exclusive BF in the sixth month and the first year of life.

## METHODS

This was a prospective, longitudinal, observational study of 90 infants diagnosed with T21. It was approved by the Research Ethics Committee of the Federal University of Minas Gerais (UFMG) under approval number 5,905,251, CAAE 66340922.8.0000.5149. Recruitment was carried out among infants participating in the outreach program named “Multidisciplinary approach to orofacial hypotonia and tongue protrusion in infants with Down syndrome” at the UFMG School of Dentistry. All parents or legal guardians signed an informed consent form.

The study included infants with a corrected age of up to 1 year and diagnosed with T21 and excluded those with ankyloglossia, contraindications for BF, for example, HIV-positive, galactosemia, and other associated syndromes and/or orofacial malformations.

The study included the following procedures to meet its objectives: clinical history survey, assessment of habitual lip and tongue posture, and assessment of the duration of exclusive and supplemented BF.

### Clinical history survey

Clinical conditions were investigated through analysis of medical history records obtained from parents or guardians of children who attended the outreach program and assessments conducted by professionals working there. They used a specific protocol developed by the authors to obtain information on sex, chronological age, gestational age, presence of tongue diastasis, and general health conditions (lung and/or heart disease). General health conditions were also approached in the 6-month and 1-year interviews.

### Assessment of habitual lip and tongue posture

The habitual lip and tongue posture was analyzed using videos of infants up to 4 months old on the day of the outreach program assessment. Therefore, only 33 infants had their videos analyzed. This age range was chosen because the first 4 months are a period of reflex sucking and the establishment of BF. Including more mature infants in this initial phase would have biased the research, as it is believed that muscle tone in non-breastfed infants may decrease over time. The aim was to assess the infants' innate abilities regarding the habitual posture of orofacial structures at a time when they may not yet have developed compensations or loss of tone related to sucking.

The habitual lip and tongue posture was assessed by analyzing a 5-minute video of the face, recorded during the infant's evaluation in the outreach program. The infant was placed on the caregiver’s lap, who was instructed not to interfere with the recordings. Age-appropriate toys were provided to keep the infant distracted.

Two researchers analyzed the videos frame by frame independently. The child's tongue posture in each frame was classified as: I) within the oral cavity (tongue behind the lower alveolar ridge); II) between the alveolar ridges (tongue over the lower alveolar ridge and behind the lower lip); III) over the lower lip (tongue touching the lower lip); IV) severe protrusion in relation to the lower lip (tongue protruded over the lower lip, with the apex exceeding the anterior limit of the lower lip)^(15)^. Lip posture was classified as: I) closed (contact between the lower and upper lips throughout the entire length of the lip rim); II) parted (contact between the upper and lower lips only near the corners of the mouth); or III) open (no contact between the lower and upper lips)^(15)^. The number of seconds the infant remained in each classification of habitual lip and tongue posture was recorded, with the posture adopted for the longest duration being considered predominant. Moments when the infant smiled or vocalized were not included in the analysis. To increase data reliability, a second researcher was trained and then performed an agreement analysis on 20% of the videos (intrarater agreement). In addition, the first researcher analyzed 20% of the videos 6 months after her first analysis (interrater agreement). The Kappa coefficient was calculated to verify agreement.

### Survey on exclusive and supplemented breastfeeding

The duration of exclusive and supplemented BF and the difficulties of the infant and mother in the process were assessed with a questionnaire, via telephone contact.

The first interview was conducted when the infant was 6 months or older, as this is the WHO's recommended period for exclusive BF. Furthermore, because the mother had been breastfeeding for longer, she already had a better ability to observe the BF difficulties and potentials than in the initial months of assessment. Ninety mothers participated in this first interview and completed a questionnaire on BF data in the sixth month of their infants' lives.

The questionnaire was developed based on other ones used in speech-language-hearing BF research^(16,17)^. Its questions approached the baby's clinical history, current feeding style, duration of BF and exclusive BF, reasons for discontinuing exclusive BF, use of formula, whether breast milk was offered in the first hour of life, and BF difficulties. It also had questions about the time of T21 diagnosis, BF guidance to the mother, whether she observed extraoral milk spillage and tongue projection during BF, and whether she used or had used a pacifier or silicone nipple shield^(17)^.

The second interview was conducted when the infant was 1 year old to monitor supplemental BF. The questionnaire approached the presence of BF, lung and/or heart disease, and pacifier use during the first year. This interview was conducted only with caregivers of infants who had already turned 1 year old by the final data collection date. Thus, only 75 mothers of infants with T21 participated.

### Data analysis methodology

The research response variables were exclusive BF in the sixth month and BF in the sixth and twelfth months. The explanatory variables were the infant’s sex, clinical conditions (prematurity, length of hospital stay, associated comorbidities, tongue diastasis, and oral habits), the mothers’ self-reported difficulties, use of formula, BF in the first hour of life, time of T21 diagnosis, BF guidance during prenatal care, and orofacial myofunctional conditions (habitual tongue and lip posture). The other variables were used to characterize the sample and discuss the associations.

Descriptive data analysis was performed using frequency distributions of categorical variables and measures of central tendency and variability of continuous variables. Pearson's chi-square test was used to assess associations between variables, with a 5% significance level.

Intrarater and interrater comparisons were also performed (Table 1). The intrarater comparison was classified as almost perfect, while the interrater comparison was classified as substantial, according to the criteria of Landis and Koch (1977)^(18)^.

**Table 1:** Intrarater and interrater comparison of the classification of habitual tongue and lip posture

Variable	Intrarater comparison		Interrater comparison	
	kappa	Classification	kappa	Classification
Habitual lip posture	1.0	Almost perfect	0.7667	Substantial
Habitual tongue posture	1.0	Almost perfect	0.7407	Substantial

Kappa test

**Caption:** Classification: slight (0 to 0.2); fair (0.21 to 0.40); moderate (0.41 to 0.60); substantial (0.61 to 0.80); and almost perfect (0.81 to 1.0)^(18)^

## RESULTS

Ninety infants diagnosed with T21 participated in the study, with a mean age of 8 months (SD=5.9) at the first interview. The duration of BF was 69 days (SD = 84, minimum of 0 and maximum of 365 days), and that of exclusive BF was 61.8 days (SD = 75.5, minimum of 0 and maximum of 180 days). The mean length of hospital stay after birth until the first year of life was 26.4 days (SD = 49.9, minimum of 0 and maximum of 300 days). The mean duration of pacifier use was 1.5 months (SD = 2.4).

Table 2 presents the analysis of the association between BF (in the sixth and first years of life) and the explanatory clinical variables. The prevalence of BF and exclusive BF in the sixth month was higher in boys and in babies who were breastfed in the first hour of life. The prevalence of BF in the first year was higher among babies who were not formula-fed and who were breastfed in the first hour of life. Lung disease was associated with BF in the first year.

**Table 2:** Association analysis between exclusive and supplemented breastfeeding in the sixth month and breastfeeding in the twelfth month and the explanatory clinical variables

Variable	EBF in the sixth month (n=90)			BF in the sixth month (n=90)			BF in the first year (n=75)		
	Yes n (%)	No n (%)	p-value	Yes n (%)	No n (%)	p-value	Yes n (%)	No n (%)	*p-value
Sex									
Females	5 (21.7)	32 (47.8)	0.029*	16 (29.1)	21 (61.8)	0.002*	7 (26.9)	25 (51.0)	0.104
Males	18 (78.3)	35 (52.2)		39 (70.9)	13 (38.2)		19 (73.1)	24 (49.0)	
Prematurity									
Yes	4 (17.4)	25 (37.3)	0.078	15 (27.3)	14 (41.2)	0.174	7 (26.9)	19 (38.8)	0.313
No	19 (82.6)	42 (62.7)		40 (72.7)	20 (58.8)		19 (73.1)	30 (61.2)	
Hospitalization (surveyed in the sixth month)									
Yes	16 (69.6)	54 (80.6)	0.272	41 (74.5)	29 (85.3)	0.229	18 (69.2)	40 (81.6)	0.458
No	7 (30.4)	13 (19.4)		14 (25.5)	5 (14.7)		8 (30.8)	9 (18.4)	
Length of hospital stay (surveyed in the sixth month)									
Never	7 (30.4)	13 (19.5)	0.100	14 (25.5)	5 (14.7)	0.258	8 (30.8)	9 (18.4)	0.524
Up to 30 days	15 (65.2)	38 (56.7)		33 (60.0)	20 (58.8)		15 (57.7)	28 (57.1)	
Over 30 days	1 (4.4)	16 (23.8)		8 (14.5)	9 (26.5)		3 (11.5)	12 (24.5)	
Gastroesophageal reflux (surveyed in the sixth month)									
Yes	7 (30.4)	22 (32.8)	0.719	17 (30.9)	12 (35.3)	0.506	8 (30.8)	17 (34.7)	0.897
No	15 (65.2)	44 (65.7)		36 (65.5)	22 (64.7)		17 (65.4)	31 (63.3)	
Not reported	1 (4.4)	1 (1.5)		2 (3.6)	0 (0.0)		1 (3.8)	1 (2.0)	
Thumb sucking (surveyed in the sixth month)									
Yes	7 (30.4)	20 (29.8)	0.948	18 (32.7)	9 (26.5)	0.797	6 (23.1)	15 (30.6)	0.773
No	15 (65.2)	45 (67.2)		35 (63.7)	24 (70.6)		19 (73.1)	32 (65.3)	
Not reported	1 (4.4)	2 (3.0)		2 (3.6)	1 (2.9)		1 (3.8)	2 (4.1)	
Lingual diastasis									
Yes	5 (28.8)	16 (28.1)	0.751	11 (24.4)	9 (31.0)	0.751	6 (30.0)	13 (30.2)	0.652
No	13 (72.2)	41 (71.9)		34 (75.6)	20 (69.0)		14 (70)	30 (69.8)	
Pulmonary disease (surveyed in the sixth month and in the first year)									
Yes	4 (17.4)	5 (7.5)	0.171	7 (12.7)	2 (5.9)	0.298	10 (38.5)	12 (24.5)	0.017*
No	19 (82.6)	62 (92.5)		48 (87.3)	32 (94.1)		16 (61.5)	37 (75.5)	
Heart disease (surveyed in the sixth month and in the first year)									
Yes	2 (8.7)	14 (20.9)	0.187	9 (16.4)	7 (20.6)	0.614	4 (15.4)	12 (24.5)	0.114
No	21 (91.3)	53 (79.1)		46 (83.6)	27 (79.4)		22 (84.6)	37 (75.5)	
Formula use									
Yes	1 (4.3)	65 (97.0)	<0.001*	31 (56.4)	34 (100.0)	<0.001*	9 (34.6)	45 (91.8)	<0.001*
No	22 (95.7)	2 (3.0)		24 (43.6)	0 (0.0)		17 (65.4)	4 (8.2)	
Breast milk offered in the first hour									
Yes	16 (69.6)	27 (40.3)	0.015*	32 (58.2)	11 (32.4)	0.018*	20 (76.9)	19 (38.8)	0.001*
No	7 (30.4)	40 (59.7)		23 (41.8)	23 (67.6)		6 (23.1)	30 (61.2)	
Trisomy 21 diagnosed in prenatal care									
Yes	5 (21.7)	27 (40.3)	0.109	19 (34.5)	12 (35.3)	0.943	8 (30.8)	16 (32.7)	0.285
No	18 (78.3)	40 (59.7)		36 (65.5)	22 (64.7)		18 (69.2)	33 (67.3)	
Breastfeeding guidelines received during prenatal care									
Yes	2 (8.7)	17 (25.4)	0.091	10 (18.2)	9 (26.5)	0.354	5 (19.2)	9 (18.4)	0.444
No	21 (91.3)	50 (74.6)		45 (81.8)	25 (73.5)		21 (80.8)	40 (81.6)	

Pearson's chi-square test

* Significant p-value ≤ 0.05

**Caption:** EBF = exclusive breastfeeding; BF = breastfeeding; n = absolute frequency; % = relative frequency

Table 3 presents the analysis of the association between BF (in the sixth month, exclusive or not, and in the twelfth month) and the explanatory variables related to the child's and mother's self-reported BF difficulties and oral habits. There were associations between maternal reports of the child's difficulty being breastfed and her own difficulty breastfeeding and the presence of BF in the sixth month; between reports of difficulty latching on and the presence of BF in the sixth month; and between low milk production and the absence of BF in the twelfth month. Children who did not use a pacifier had a higher prevalence of exclusive BF in the sixth month and of BF at 12 months of age.

**Table 3:** Association analysis between exclusive and supplemented breastfeeding in the sixth month and breastfeeding in the twelfth month and the explanatory variables related to the child’s and the mother’s difficulties reported by the mother and oral habits

Variable	EBF in the sixth month (n=90)			BF in the sixth month (n=90)				BF in the first year (n=75)		
	Yes n (%)	No n (%)	p-value	Yes n (%)	No n (%)	p-value	Yes n (%)		No n (%)	*p-value
Child’s breastfeeding difficulties										
Yes	18 (78.3)	43 (64.2)	0.212	45 (81.8)	16 (47.1)	0.001*	21 (80.8)		28 (57.1)	0.062
No	5 (21.7)	24 (35.8)		10 (18.2)	18 (52.9)		5 (19.2)		21 (42.9)	
Sucking difficulties										
Yes	9 (39.1)	25 (37.3)	0.877	22 (40.0)	12 (35.3)	0.657	11 (42.3)		19 (38.8)	0.596
No	14 (60.9)	42 (62.7)		33 (60.0)	22 (64.7)		15 (57.7)		30 (61.2)	
Latching difficulties										
Yes	8 (34.8)	21 (31.3)	0.761	24 (43.6)	5 (14.7)	0.005*	10 (38.5)		11 (22.4)	0.059
No	15 (65.2)	46 (68.7)		31 (56.4)	29 (85.3)		16 (61.5)		38 (77.6)	
Excessive sleepiness										
Yes	5 (21.7)	8 (11.9)	0.249	11 (20.0)	2 (5.9)	0.067	3 (11.5)		3 (6.1)	0.067
No	18 (78.3)	59 (88.1)		44 (80.0)	32 (94.1)		23 (88.5)		46 (93.9)	
Tiredness										
Yes	4 (17.4)	12 (17.9)	0.955	13 (23.6)	3 (8.8)	0.077	5 (19.2)		7 (14.3)	0.533
No	19 (82.6)	55 (82.1)		42 (76.4)	31 (91.2)		21 (80.8)		42 (85.7)	
Cough or choking										
Yes	2 (8.7)	8 (11.9)	0.669	9 (16.4)	1 (2.9)	0.051	3 (11.5)		3 (6.1)	0.086
No	21 (91.3)	59 (88.1)		46 (83.6)	33 (97.1)		23 (88.5)		46 (93.9)	
Mother’s breastfeeding difficulties										
Yes	11 (47.8)	31 (46.3)	0.897	30 (54.5)	11 (32.4)	0.041*	10 (38.5)		24 (49.0)	0.584
No	12 (52.2)	36 (53.7)		25 (45.5)	23 (67.6)		16 (61.5)		25 (51.0)	
Breast pain or soreness										
Yes	4 (17.4)	4 (6.0)	0.097	7 (12.7)	1 (3.0)	0.117	2 (7.7)		3 (6.1)	0.247
No	19 (82.6)	63 (94.0)		48 (87.3)	33 (97.0)		24 (92.3)		46 (93.9)	
Low milk production										
Yes	19 (82.6)	16 (23.9)	0.518	11 (20)	8 (23.5)	0.693	3 (11.5)		16 (32.7)	0.032*
No	4 (17.4)	51 (76.1)		44 (80)	26 (76.5)		23 (88.5)		33 (67.3)	
Extraoral spillage during breastfeeding										
Yes	9 (39.1)	28 (41.8)	0.823	25 (45.5)	12 (35.3)	0.345	10 (38.5)		20 (40.8)	0.874
No	14 (60.9)	39 (58.2)		30 (54.5)	22 (64.7)		16 (61.5)		29 (59.2)	
Tongue protruding during breastfeeding										
Yes	8 (34.8)	28 (41.8)	0.554	24 (43.6)	12 (35.3)	0.436	13 (50.0)		20 (40.8)	0.166
No	15 (78.3)	39 (58.2)		31 (56.4)	22 (64.7)		13 (50.0)		29 (59.2)	
Pacifier (surveyed in the sixth month and in the first year)										
Yes	2 (8.7)	25 (37.3)	0.010*	13 (23.6)	13 (38.2)	0.141	0 (0.0)		15 (30.6)	<0.001*
No	21 (91.3)	42 (62.7)		42 (76.4)	21 (61.8)		26 (100.0)		34 (69.4)	
Silicone nipple shield (surveyed in the sixth month)										
Yes	4 (17.4)	19 (28.4)	0.298	15 (27.3)	8 (23.5)	0.695	5 (19.2)		11 (22.4)	0.116
No	19 (82.6)	48 (71.6)		40 (72.7)	26 (76.5)		21 (80.8)		38 (77.6)	

Pearson's chi-square test

* Significant p-value ≤ 0.05

**Caption:** EBF = exclusive breastfeeding; BF = breastfeeding; n = absolute frequency; % = relative frequency

The habitual lip and tongue posture predominantly adopted by infants in the assessment (up to 4 months) was not associated with the BF situation in the sixth or twelfth month (Table 4).

**Table 4:** Association of habitual tongue and lip posture in the first assessment with exclusive and supplemented breastfeeding in the sixth month and with breastfeeding in the twelfth month

Variable	EBF in the sixth month (n=33)			BF in the sixth month (n=33)			BF in the first year (n=20)		
	Yes n (%)	No n (%)	p-value	Yes n (%)	No n (%)	p-value	Yes n (%)	No n (%)	*p-value
Habitual lip posture									
Open lips	2 (28.6)	17 (65.4)	0.072	14 (58.4)	5 (55.6)	0.968	6 (85.7)	8 (61.5)	0.193
Parted lips	3 (42.8)	8 (30.8)		8 (33.3)	3 (33.3)		1 (14.3)	1 (7.7)	
Closed lips	2 (28.6)	1 (3.8)		2 (8.3)	1 (11.1)		0 (0)	4 (30.8)	
Habitual tongue posture									
Within the oral cavity	4 (57.1)	7 (26.9)	0.313	8 (33.3)	3 (33.3)	0.907	2 (28.6)	4 (30.8)	0.326
Between alveolar ridges	2 (28.6)	11 (42.3)		9 (37.5)	4 (44.5)		1 (14.3)	6 (46.2)	
Over the lower lip	1 (14.3)	8 (30.8)		7 (29.2)	2 (22.2)		4 (57.1)	3 (23)	

Pearson's chi-square test

* Significant p-value ≤ 0.05

**Caption:** EBF = exclusive breastfeeding; BF = breastfeeding; n = absolute frequency; % = relative frequency

## DISCUSSION

This study aimed to investigate the duration of BF and exclusive BF in infants with T21 and to verify the association between clinical and orofacial myofunctional conditions and the presence of BF and exclusive BF in the sixth month and in the first year of life. This study, as well as others^(6,7,10,19)^, found that infants with T21 had a short duration of BF – 69 days (SD = 84, minimum of 0 and maximum of 365 days) – and exclusive BF – 61.8 days (SD = 75.5, minimum of 0 and maximum of 180 days). A Chilean study^(10)^ found a frequency of 46.6% of exclusive BF at 6 months in infants with T21. A literature review indicated a variation of 31.6 to 55.4% of infants with T21 who received exclusive BF and 23.3 to 50% who were never breastfed^(7)^. This differs from the data found in the present study, in which 25.6% of infants were exclusively breastfed at 6 months, and 36.7% were never breastfed. Divergences between studies may be due to population characteristics and socioeconomic and sociodemographic differences, as they were conducted in different countries. In the study conducted in Chile^(10)^, for example, 75% of participants had maternity leave of 6 months or more, which may have positively influenced the duration of exclusive BF.

Regarding BF duration, the researchers of an Italian study^(6)^ found that infants with T21 were breastfed for 54 days, while infants without the syndrome were breastfed for up to 164 days. Thus, the findings of the present study agree with the literature regarding a shorter BF than recommended.

In the analysis of clinical variables, males had a higher prevalence of BF or exclusive BF in the sixth month. No studies were found that conducted this investigation with infants with T21. A Brazilian study found that male infants without the syndrome were breastfed for longer than females^(20)^. An Indian study also found a shorter BF in girls^(21)^. On the other hand, a Senegalese study found that the duration of exclusive BF was shorter in male than in female infants^(22)^. No possible explanations for this result were found, but it is believed that comparisons between the sexes regarding growth curve and number of hospitalizations could provide additional information on why boys were breastfed for longer.

BF in the twelfth month of life was associated with lung disease, contradicting the literature^(23)^. A systematic review reports that non-BF practices pose a significant risk for acute respiratory infection and hospitalization, while exclusive BF for 4 to 6 months significantly reduced hospitalization, length of stay, need for supplemental oxygen, and intensive care unit admission^(23)^. It is believed that other variables not investigated in this study, linked to the presence of lung diseases, may have been confounding factors (craniofacial characteristics, respiratory muscle hypotonia, child's posture, type of lung complications, and so forth). Furthermore, lung diseases constitute a broad category of clinical conditions, including asthma, bronchitis, bronchiolitis, and pneumonia, among others, which were not differentiated in this study. Therefore, the relationship between BF and lung diseases in infants with T21 needs further investigation.

Formula use was associated with not being on BF or exclusive BF in the sixth and twelfth months of life. This finding agrees with the literature^(24,25)^. By offering formula instead of the mother's breast, there is less suction stimulation at the breast, which leads to decreased milk production and culminates in early weaning^(24)^. Furthermore, the formula is offered in a bottle, which requires less effort for sucking, and it is often difficult to stop using this utensil to return to BF, once the infant becomes accustomed to it^(25)^. A study^(24)^ indicates that the use of artificial nipples can cause spacing between feedings, impacting milk production, leading to nipple and flow confusion and, consequently, early weaning.

The results suggest that BF in the first hour of life is important for maintaining BF and exclusive BF until the sixth month and BF until the twelfth month, corroborating previous research that indicates that early contact between the newborn and the mother's breast in the first hour of life influences the initiation, maintenance, and duration of exclusive BF^(26)^. Furthermore, skin-to-skin contact in the first hour of life favors BF success, including exclusive BF for a longer period^(26)^. Nevertheless, a Swedish study indicated, based on reports from mothers of babies with T21, that health professionals did not encourage BF during the first hour of life, regardless of the babies’ clinical condition^(8)^. The present research did not investigate whether the absence of BF in the first hour was due to the baby's clinical condition, which is a limitation of the study. It is known that T21 can be associated with several comorbidities^(4)^, which this study did not investigate, although they can hinder BF^(9)^. These include the number of hospitalizations, surgeries, and other hospital procedures that separate the dyad. A Puerto Rican study with mothers of babies with T21 found that 50% of them were unable to stay with their babies during their hospital stay^(11)^.

The analysis of variables related to the mother's report found an association between maternal perception of infant BF difficulty and the presence of BF in the sixth month. This association can be justified by the fact that the mothers who reported this difficulty were breastfeeding. Thus, they perceived the infant’s real difficulties. This perception was linked to the first 6 months, possibly because they are the most difficult in this process^(27)^. The literature points to the relationship between primiparity and maternal BF difficulty^(28)^. However, the present study did not collect this variable. Among the difficulties studied, latching seems to be the main issue, given the association also found between BF in the sixth month and difficulties related to latching. The literature justifies the difficulty with latching in T21 by the hypotonia of the orbicularis oris and buccinator muscles, a characteristic of T21^(1)^, which compromises the generation of negative intraoral pressure, important for milk extraction^(5,13)^. Furthermore, in the process of extracting milk from the breast, there is great participation of the jaw elevator muscles, which may also be hypotonic in infants with the syndrome^(5)^.

Mothers who supplemented BF until the sixth month of their babies' lives reported their BF difficulties in greater proportion, such as breast pain/soreness and decreased milk production. Again, these can be justified by the fact that they were breastfeeding and noticed such difficulties. In this context, low milk production was associated with the absence of BF in the twelfth month, which is due to the lack of stimulation of the baby's sucking on the mother's breast, consequently decreasing milk production^(24)^.

Pacifier use had a direct impact on not being on exclusive BF at 6 months or BF at 12 months. This finding can be explained by the fact that pacifier use reduces BF frequency, thus compromising breast milk production and, consequently, posing a risk of BF interruption. An epidemiological study comparing pacifier use and BF duration found that infants who did not use the device were four times more likely to continue breastfeeding until 6 months than those who used it full-time^(29)^. In 2017, an observational study identified an association between pacifier use and exclusive BF interruption, concluding that pacifier use is a risk factor for the latter in infants under 6 months old^(30)^. Furthermore, a meta-analysis identified that pacifier use doubled the risk of interrupting BF. It has also been reported that the pacifier is considered a marker of BF difficulties and decreases maternal motivation to breastfeed^(31)^.

Habitual lip and tongue posture was expected to be associated with the presence of BF and exclusive BF. This association may not have been found due to the sample size for analysis and the duration of the videos (only 5 minutes), which may not reflect the posture adopted by infants most of the time, portraying only a specific moment. However, although not statistically significant, infants with a habitual closed-lip posture in the first 4 months were more commonly on exclusive BF at 6 months, unlike those with open lips. This finding may be explained by the hypotonia of the orbicularis oris muscle, present in cases of open-lip posture, which directly impacts latching on, influencing the duration and maintenance of exclusive BF until the sixth month of life^(2)^. Following the same reasoning, a higher proportion of tongues were positioned within the oral cavity among infants who were breastfed at six months, either exclusively or not. The tongue was considered flat with a rounded tip (resting on the floor of the mouth), elevated (resting on the palate), or invisible (closed lips). These positions are associated with better tongue muscle tone compared to the tongue whose habitual position is between the alveolar ridges or over the lower lip. The literature indicates that infants without lingual frenulum changes tend to maintain the habitual elevated tongue posture^(32)^. Thus, an elevated tongue attached to the palate has been considered the normal standard^(32,33)^. However, regarding tongue position, this study focused only on its anteroposterior relationship – i.e., how forward the tongue was within the oral cavity, since lingual protrusion is a characteristic generally reported in individuals with T21.

The study in question confirmed the hypothesis that BF duration is associated with formula use, maternal and infant difficulties, and pacifier use. Furthermore, the results provide a better understanding of the factors that influence BF in infants with T21, given that studies on this specific population are still scarce in the literature. The impacts of these infants' comorbidities and oral habits directly affect early weaning and, consequently, can influence the development of orofacial structures, in addition to chewing, sucking, and swallowing. Successful development of these functions ensures improved tone.

The study's limitations include the small number of participants, the lack of assessment of respiratory pattern, and the fact that the information was based on questionnaires that considered maternal perceptions of the aspects studied. Nonetheless, maternal perception is important to understand the main factors that influence the duration and maintenance of BF and exclusive BF. The subjectivity related to the analysis of habitual lip and tongue posture can also be cited as a limitation. To minimize the subjectivity inherent in data collection, a quantitative analysis based on videos was performed to assess habitual posture, verifying intrarater and interrater agreement, which can be considered a strength of the study. The fact that the intrarater comparison was almost perfect and the interrater comparison was substantial demonstrates good reproducibility in the video analysis, increasing the reliability of these analyses. The findings of this study may be useful for healthcare professionals in better guiding parents and guardians of infants with T21 to maintain BF in this population.

## CONCLUSION

This study found a low prevalence of exclusive BF in the sixth month and BF in the sixth and twelfth months in infants with T21.

There were associations between male infants and BF and exclusive BF in the sixth month; between BF in the first hour of life and exclusive BF in the sixth month and BF in the sixth and twelfth months; between formula use and the absence of exclusive BF in the sixth month and BF in the sixth and twelfth months; between lung disease and BF in the twelfth month; and between pacifier use and the absence of exclusive BF in the sixth month and BF in the twelfth month.

Mothers who were breastfeeding in the sixth month reported BF difficulties for both the infant and themselves more frequently, with an association between reports of difficulty in latching on and BF in the sixth month and between reports of low milk production and the absence of BF in the twelfth month.
