# Supplementary material for: Factors associated with breastfeeding in infants with trisomy 21
Source: Codas. 2025 Dec 1;37(6):e20240267. doi: 10.1590/2317-1782/e20240267en (PMC12704607; doi:10.1590/2317-1782/e20240267en)
Supplement: Translation

# Translation: Fatores associados à amamentação em lactentes com trissomia do cromossomo 21

**DOI:** 10.1590/2317-1782/e20240267pt

**Authors:** Larissa Melgaço Campos, Ana Elisa Ribeiro Fernandes, Andréa Rodrigues Motta, Renata Maria Moreira Moraes Furlan

**Affiliations:** 1 Programa de Pós-graduação em Ciências Fonoaudiológicas, Universidade Federal de Minas Gerais – UFMG - Belo Horizonte (MG), Brasil.; 2 Departamento de Pediatria, Faculdade de Medicina, Universidade Federal de Minas Gerais – UFMG - Belo Horizonte (MG), Brasil.; 3 Departamento de Fonoaudiologia, Universidade Federal de Minas Gerais – UFMG - Belo Horizonte (MG), Brasil.

**Keywords:** Aleitamento Materno, Síndrome de Down, Sistema Estomatognático, Fonoaudiologia, Língua

## Abstract

**Objetivo:**

Pesquisar a duração do aleitamento materno exclusivo (AME) e do aleitamento materno (AM) em lactentes com T21 e verificar a associação entre as condições clínicas e miofuncionais orofaciais com a presença de AME e de AM no sexto mês e no primeiro ano de vida.

**Método:**

Estudo observacional longitudinal prospectivo, com 90 lactentes com T21. Os pais responderam dois questionários acerca das condições clínicas e da amamentação, um no 6º e outro no 12º mês do lactente. A postura habitual de lábios e língua foi obtida por meio de vídeos e a história clínica por consulta ao prontuário. A presença de AME e de AM no 6º e no 12º mês foram associadas às condições clínicas e miofuncionais orofaciais por meio do teste Qui-quadrado de Pearson, com 5% de significância.

**Resultados:**

Houve associação entre sexo masculino e presença de AME/AM ao sexto mês; uso de fórmula e ausência de AM; amamentar na primeira hora e presença de AM no primeiro ano; doença pulmonar e estar em AM no primeiro ano; dificuldade na amamentação com presença do AM no sexto mês; presença de AM no sexto mês e dificuldade na pega; baixa produção láctea e ausência de AM no primeiro ano; uso de chupeta e ausência de AME no sexto mês e AM no primeiro ano.

**Conclusão:**

Sexo, doença pulmonar, uso de fórmula, amamentação na primeira hora de vida, dificuldade autorrelatada para amamentar, baixa produção láctea e uso de chupeta foram fatores associados à amamentação em lactentes com T21.

## INTRODUÇÃO

A trissomia do cromossomo 21 (T21) ou síndrome de Down consiste em uma condição genética, causada pela triplicação do cromossomo 21, tendo como uma de suas manifestações clínicas a hipotonia muscular^(1)^, o que inclui as estruturas orofaciais, como lábios e língua. Essa hipotonia altera a postura habitual dessas estruturas e prejudica as funções estomatognáticas de sucção, mastigação e deglutição, podendo levar a dificuldades no processo de aleitamento materno (AM) e, posteriormente, na introdução alimentar^(2)^. Ademais, bebês com T21 podem apresentar comorbidades associadas que também podem influenciar nesses processos, como cardiopatias congênitas^(3)^, com aumento da demanda metabólica e taquidispneia durante a sucção, alterações do sistema digestório e respiratório, entre outras alterações^(4)^.

Lactentes com T21 podem apresentar dificuldade na amamentação devido à hipotonia muscular^(5)^. Na literatura há relatos sobre duração menor do AM em lactentes com T21^(6,7)^, porém há poucos estudos que abordam as causas das dificuldades na amamentação e do desmame precoce, podendo estar associadas às especificidades da síndrome e às diversas comorbidades associadas. Ademais, observa-se uma falta de preparo dos profissionais de saúde para lidar com a amamentação em situações atípicas, podendo levar à suspensão do AM pela falta de informação^(8)^.

Estudos apontam que lactentes com T21 amamentam com menos frequência, quando comparados com populações típicas devido a condições clínicas que são vistas como dificultadoras do processo de amamentação^(9)^. Um estudo^(6)^, realizado na Itália, revelou que a duração média de amamentação entre crianças com T21 foi de 54 dias, comparada a 164 dias no grupo controle. Outro estudo, conduzido no Chile, verificou maior frequência de aleitamento materno exclusivo (AME) até o sexto mês (46,6%), com prevalência de 71,2% de AM, sendo que 96% das mães amamentaram por, pelo menos, um mês^(10)^. Em contrapartida, uma pesquisa^(11)^ encontrou, em Porto Rico, que 84,6% das mães de lactentes com T21 optaram por não amamentar ou descontinuaram o aleitamento devido à dificuldade de sucção do lactente e por não receberem orientações sobre aleitamento durante a internação.

A Organização Mundial de Saúde (OMS) recomenda o AME até os seis meses de vida, pelo fato de o leite materno suprir todas as necessidades nutricionais do bebê nessa idade^(12)^. Outrossim, o AM contribui para o desenvolvimento das estruturas miofuncionais orofaciais^(13)^ que, no caso da T21, estão hipotônicas, o que torna a amamentação ainda mais benéfica^(13)^. Para tanto, torna-se necessário um maior conhecimento e preparo dos profissionais da saúde para orientar as mães desses bebês quanto ao AM, visto que essa etapa inicial, se não cumprida com sucesso, pode afetar as etapas posteriores, como a aceitação dos primeiros alimentos ofertados^(14)^.

Diante disso, o presente estudo tem por objetivo pesquisar a duração do AME e do AM em lactentes com T21 e verificar a associação entre as condições clínicas e miofuncionais orofaciais com a presença de AME e de AM no sexto mês e no primeiro ano de vida.

## MÉTODO

Trata-se de um estudo observacional longitudinal prospectivo, realizado com 90 lactentes com diagnóstico de T21. O estudo foi aprovado pelo Comitê de Ética em Pesquisa da Universidade Federal de Minas Gerais (UFMG), número do parecer 5.905.251, CAAE 66340922.8.0000.5149. O recrutamento foi realizado dentre os lactentes que participavam do Projeto de Extensão “Abordagem multidisciplinar da hipotonia orofacial e protrusão lingual de bebês com Síndrome de Down”, na Faculdade de Odontologia da UFMG. Todos os pais ou responsáveis legais assinaram o Termo de Consentimento Livre e Esclarecido.

Foram incluídos os lactentes com idade corrigida de até 1 ano, com diagnóstico de T21. Foram excluídos os lactentes com anquiloglossia, aqueles com contraindicação para o AM, por exemplo, HIV positivo, galactosemia e com outras síndromes e/ou más-formações orofaciais associadas.

Para atender aos objetivos propostos, o estudo contou com os seguintes procedimentos: pesquisa do histórico clínico, avaliação da postura habitual de lábios e de língua e avaliação da duração do AME e do aleitamento complementado.

### Pesquisa do histórico clínico

As condições clínicas foram investigadas por meio da análise dos registros da anamnese realizada com os pais ou responsáveis das crianças atendidas no projeto de extensão e avaliação realizadas pelos profissionais que lá atuam, utilizando um protocolo específico, elaborado pelos autores. Foram obtidas informações sobre sexo, idade cronológica, idade gestacional, presença de diástase lingual e condições gerais de saúde (doença pulmonar e/ou cardíaca). As condições gerais de saúde também foram questionadas na entrevista do sexto mês e de um ano.

### Avaliação da postura habitual de lábios e de língua

A análise da postura habitual de lábios e de língua foi realizada a partir dos vídeos de lactentes que estavam com idade de até 4 meses no dia da avaliação no projeto de extensão. Dessa forma, apenas 33 lactentes tiveram os vídeos analisados. Esse corte de faixa etária foi realizado pelo fato de os primeiros quatro meses constituírem um período de sucção reflexa e de estabelecimento da amamentação. A inclusão de lactentes mais maduros nessa primeira fase traria um viés à pesquisa, pois acredita-se que o tônus muscular de lactentes não amamentados possa diminuir com o passar do tempo. Pretendeu-se, assim, avaliar as habilidades natas dos lactentes, referentes à postura habitual das estruturas orofaciais, em um momento em que possivelmente ainda não desenvolveram compensações ou perdas de tônus relacionadas ao modo de sucção.

A avaliação da postura habitual de lábios e de língua foi realizada por meio da análise de vídeo da face com duração de cinco minutos, gravado durante a avaliação do lactente no projeto de extensão. O lactente foi posicionado no colo do responsável, o qual foi orientado a não interferir nas gravações. Brinquedos apropriados à idade foram oferecidos para manter o lactente distraído.

Os vídeos foram analisados, quadro a quadro, por duas pesquisadoras de forma independente. Em cada quadro, a postura de língua da criança foi classificada como: I) dentro da cavidade oral (língua atrás do rolete gengival inferior); II) entre os roletes gengivais (língua sobre o rolete gengival inferior e atrás do lábio inferior); III) sobre o lábio inferior (língua tocando o lábio inferior); IV) protrusão severa em relação ao lábio inferior (língua protruída, sobre o lábio inferior, com o ápice ultrapassando o limite anterior do lábio inferior)^(15)^. A postura de lábios foi classificada como: I) fechados (presença de contato entre os lábios inferior e superior em toda a extensão da rima labial); II) semiabertos (contato entre os lábios superior e inferior apenas próximo às comissuras labiais); ou III) abertos (não há contato entre lábio inferior e superior)^(15)^. Foram contabilizados os segundos em que o lactente permaneceu em cada classificação da postura habitual de lábios e de língua, sendo considerada como predominante a postura adotada por maior tempo. Os momentos em que o lactente sorriu ou vocalizou não foram considerados na análise. Para aumentar a confiabilidade dos dados, uma segunda pesquisadora, após treinamento, realizou a análise de concordância de 20% dos vídeos (concordância intra-avaliador). Além disso, a primeira pesquisadora realizou a análise de 20% dos vídeos, 6 meses após a sua primeira análise (concordância interavaliador). Para verificação das concordâncias foi calculado o coeficiente Kappa.

### Pesquisa do aleitamento materno exclusivo e complementado

A avaliação da duração do AME e do aleitamento complementado e das dificuldades do lactente e da mãe no processo foi realizada por meio da aplicação de questionário, via contato telefônico.

A primeira entrevista foi realizada quando o lactente se encontrava em idade a partir do sexto mês, pelo fato de esse período ser recomendado pela OMS para o AME. Ademais, nesse período, pelo fato de a mãe estar amamentando há mais tempo, ela já apresentava melhor capacidade de observação das dificuldades e potencialidades do processo de amamentação quando comparado aos meses iniciais de avaliação. Nessa primeira entrevista participaram 90 mães para responder ao questionário dos dados de aleitamento no sexto mês de vida dos lactentes em questão.

O questionário foi elaborado com base em questionários utilizados em pesquisas com a temática de AM em Fonoaudiologia^(16,17)^, sendo composto por questões sobre o histórico clínico do bebê, a forma de alimentação atual, duração do AME e do AM, motivos que levaram à interrupção do AME, uso de fórmula, se o leite materno foi ofertado na primeira hora de vida e dificuldades na amamentação. Também apresentava questões sobre a época do diagnóstico de T21, orientações recebidas sobre amamentação, se a mãe observava escape de leite extraoral e projeção de língua durante a mamada, se faz ou fez uso de chupeta e/ou bico intermediário de silicone^(17)^.

A segunda entrevista foi realizada quando o lactente se encontrava na idade de um ano, a fim de acompanhar a amamentação complementada. O questionário foi composto por questões sobre a presença do AM, presença de doença pulmonar e/ou cardíaca e se fazia uso da chupeta no primeiro ano. Essa entrevista foi realizada apenas com os responsáveis pelos lactentes que já haviam completado um ano até a data final da coleta de dados, portanto dela participaram apenas 75 mães de lactentes com T21.

### Metodologia de análise dos dados

As variáveis resposta da pesquisa foram: presença de AME no sexto mês e AM no sexto e décimo segundo meses. As variáveis explicativas foram: sexo, condições clínicas dos bebês (prematuridade, tempo de internação, comorbidades associadas, presença de diástase lingual e hábitos orais), dificuldades autorrelatadas pelas mães, uso de fórmula, aleitamento na primeira hora de vida, momento do diagnóstico da T21, recebimento de orientações sobre AM no pré-natal e condições miofuncionais orofaciais (postura habitual de língua e postura habitual de lábios). As demais variáveis coletadas foram utilizadas para caracterizar a amostra e discutir as associações encontradas.

A análise descritiva dos dados foi realizada por meio de distribuição de frequências das variáveis categóricas, medidas de tendência central e variabilidade das variáveis contínuas. Foi aplicado o teste Qui-quadrado de Pearson para verificar a associação entre as variáveis, considerando nível de significância de 5%.

Ademais, foi realizada a comparação intra e interavaliador (Tabela 1). A comparação intra-avaliador foi classificada como excelente, enquanto a comparação interavaliador classificada como substancial, de acordo com os critérios de Landis e Koch^(18)^.

**Tabela 1:** Comparação intra-avaliador e interavaliador da classificação da postura habitual de língua e de lábios

Variável	Comparação intra-avaliador		Comparação interavaliador	
	kappa	Classificação	kappa	Classificação
Postura habitual de lábios	1,0	Excelente	0,7667	Substancial
Postura habitual de língua	1,0	Excelente	0,7407	Substancial

Teste Kappa

Legenda: Classificação: fraca (0 a 0,2); razoável (0,21 a 0,40); moderada (0,41 a 0,60); substancial (0,61 a 0,80); e excelente (0,81 a 1,0)^(18)^

## RESULTADOS

Noventa lactentes com diagnóstico de T21 participaram do estudo, com média de idade de 8 meses (DP=5,9) na primeira entrevista. A duração do AM foi 69 dias (DP=84, mínimo de 0 e máximo de 365 dias) e do AME foi de 61,8 dias (DP=75,5, mínimo de 0 e máximo de 180 dias). O tempo médio de internação após o nascimento até o primeiro ano de vida foi de 26,4 dias (DP=49,9, mínimo de 0 e máximo de 300 dias). O tempo médio de uso de chupeta foi de 1,5 meses (DP=2,4).

A Tabela 2 apresenta a análise de associação entre o aleitamento materno (no sexto e no primeiro ano de vida) e as variáveis clínicas explicativas. As prevalências de AME e AM no sexto mês foram maiores em meninos e nos bebês que amamentaram na primeira hora de vida. Já a prevalência de AM no primeiro ano foi maior entre os bebês que não usavam fórmula e que tinham amamentado na primeira hora de vida. Houve associação, também, entre presença de doença pulmonar e estar em aleitamento no primeiro ano.

**Tabela 2:** Análise de associação entre o aleitamento materno exclusivo e complementado no sexto mês e o aleitamento materno no décimo segundo mês com as variáveis clínicas explicativas

Variável	AME no sexto mês (n=90)			AM no sexto mês (n=90)			AM no primeiro ano (n=75)		
	Sim n (%)	Não n (%)	Valor de p	Sim n (%)	Não n (%)	Valor de p	Sim n (%)	Não n (%)	Valor de p*
Sexo									
Feminino	5 (21,7)	32 (47,8)	0,029*	16 (29,1)	21 (61,8)	0,002*	7 (26,9)	25 (51,0)	0,104
Masculino	18 (78,3)	35 (52,2)		39 (70,9)	13 (38,2)		19 (73,1)	24 (49,0)	
Prematuridade									
Sim	4 (17,4)	25 (37,3)	0,078	15 (27,3)	14 (41,2)	0,174	7 (26,9)	19 (38,8)	0,313
Não	19 (82,6)	42 (62,7)		40 (72,7)	20 (58,8)		19 (73,1)	30 (61,2)	
Internação (pesquisada ao sexto mês)									
Sim	16 (69,6)	54 (80,6)	0,272	41 (74,5)	29 (85,3)	0,229	18 (69,2)	40 (81,6)	0,458
Não	7 (30,4)	13 (19,4)		14 (25,5)	5 (14,7)		8 (30,8)	9 (18,4)	
Tempo de internação (pesquisado ao sexto mês)									
Nunca	7 (30,4)	13 (19,5)	0,100	14 (25,5)	5 (14,7)	0,258	8 (30,8)	9 (18,4)	0,524
Até 30 dias	15 (65,2)	38 (56,7)		33 (60,0)	20 (58,8)		15 (57,7)	28 (57,1)	
Mais de 30 dias	1 (4,4)	16 (23,8)		8 (14,5)	9 (26,5)		3 (11,5)	12 (24,5)	
Refluxo gastroesofágico (pesquisado ao sexto mês)									
Sim	7 (30,4)	22 (32,8)	0,719	17 (30,9)	12 (35,3)	0,506	8 (30,8)	17 (34,7)	0,897
Não	15 (65,2)	44 (65,7)		36 (65,5)	22 (64,7)		17 (65,4)	31 (63,3)	
Não respondeu	1 (4,4)	1 (1,5)		2 (3,6)	0 (0,0)		1 (3,8)	1 (2,0)	
Sucção digital (pesquisada ao sexto mês)									
Sim	7 (30,4)	20 (29,8)	0,948	18 (32,7)	9 (26,5)	0,797	6 (23,1)	15 (30,6)	0,773
Não	15 (65,2)	45 (67,2)		35 (63,7)	24 (70,6)		19 (73,1)	32 (65,3)	
Não respondeu	1 (4,4)	2 (3,0)		2 (3,6)	1 (2,9)		1 (3,8)	2 (4,1)	
Diástase lingual									
Sim	5 (28,8)	16 (28,1)	0,751	11 (24,4)	9 (31,0)	0,751	6 (30,0)	13 (30,2)	0,652
Não	13 (72,2)	41 (71,9)		34 (75,6)	20 (69,0)		14 (70)	30 (69,8)	
Doença pulmonar (pesquisada ao sexto mês e com um ano)									
Sim	4 (17,4)	5 (7,5)	0,171	7 (12,7)	2 (5,9)	0,298	10 (38,5)	12 (24,5)	0,017*
Não	19 (82,6)	62 (92,5)		48 (87,3)	32 (94,1)		16 (61,5)	37 (75,5)	
Doença cardíaca (pesquisada ao sexto mês e com um ano)									
Sim	2 (8,7)	14 (20,9)	0,187	9 (16,4)	7 (20,6)	0,614	4 (15,4)	12 (24,5)	0,114
Não	21 (91,3)	53 (79,1)		46 (83,6)	27 (79,4)		22 (84,6)	37 (75,5)	
Uso de fórmula									
Sim	1 (4,3)	65 (97,0)	<0,001*	31 (56,4)	34 (100,0)	<0,001*	9 (34,6)	45 (91,8)	<0,001*
Não	22 (95,7)	2 (3,0)		24 (43,6)	0 (0,0)		17 (65,4)	4 (8,2)	
Leite materno ofertado na primeira hora									
Sim	16 (69,6)	27 (40,3)	0,015*	32 (58,2)	11 (32,4)	0,018*	20 (76,9)	19 (38,8)	0,001*
Não	7 (30,4)	40 (59,7)		23 (41,8)	23 (67,6)		6 (23,1)	30 (61,2)	
Diagnóstico da trissomia do 21 no pré-natal									
Sim	5 (21,7)	27 (40,3)	0,109	19 (34,5)	12 (35,3)	0,943	8 (30,8)	16 (32,7)	0,285
Não	18 (78,3)	40 (59,7)		36 (65,5)	22 (64,7)		18 (69,2)	33 (67,3)	
Orientações para amamentação recebidas no pré-natal									
Sim	2 (8,7)	17 (25,4)	0,091	10 (18,2)	9 (26,5)	0,354	5 (19,2)	9 (18,4)	0,444
Não	21 (91,3)	50 (74,6)		45 (81,8)	25 (73,5)		21 (80,8)	40 (81,6)	

Teste Qui-quadrado de Pearson

* Valor de p significante ≤0,05

Legenda: AME = aleitamento materno exclusivo; AM = aleitamento materno; n = frequência absoluta; % = frequência relativa

A Tabela 3 apresenta a análise de associação entre o aleitamento materno (no sexto mês, exclusivo ou não, e no décimo segundo mês) e as variáveis explicativas relacionadas às dificuldades da criança e da mãe em relação à amamentação autorrelatadas pela mãe, bem como os hábitos orais. Houve associação entre o relato materno quanto à dificuldade de a criança ser amamentada e dela própria para amamentar e presença do AM no sexto mês; entre relato de dificuldade na pega e presença de AM no sexto mês; e entre baixa produção láctea e ausência de AM no décimo segundo mês. Crianças que não usavam chupeta tiveram uma prevalência maior de AME no 6º mês e de AM aos 12 meses de idade.

**Tabela 3:** Análise de associação entre o aleitamento materno exclusivo e complementado no sexto mês e o aleitamento materno no décimo segundo mês com as variáveis explicativas relacionadas às dificuldades da criança e da mãe autorrelatadas pela mãe e hábitos orais

Variável	AME no sexto mês (n=90)			AM no sexto mês (n=90)				AM no primeiro ano (n=75)		
	Sim n (%)	Não n (%)	Valor de p	Sim n (%)	Não n (%)	Valor de p	Sim n (%)		Não n (%)	*Valor de p
Dificuldade da criança para amamentar										
Sim	18 (78,3)	43 (64,2)	0,212	45 (81,8)	16 (47,1)	0,001*	21 (80,8)		28 (57,1)	0,062
Não	5 (21,7)	24 (35,8)		10 (18,2)	18 (52,9)		5 (19,2)		21 (42,9)	
Dificuldade de sucção										
Sim	9 (39,1)	25 (37,3)	0,877	22 (40,0)	12 (35,3)	0,657	11 (42,3)		19 (38,8)	0,596
Não	14 (60,9)	42 (62,7)		33 (60,0)	22 (64,7)		15 (57,7)		30 (61,2)	
Dificuldade na pega										
Sim	8 (34,8)	21 (31,3)	0,761	24 (43,6)	5 (14,7)	0,005*	10 (38,5)		11 (22,4)	0,059
Não	15 (65,2)	46 (68,7)		31 (56,4)	29 (85,3)		16 (61,5)		38 (77,6)	
Sonolência excessiva										
Sim	5 (21,7)	8 (11,9)	0,249	11 (20,0)	2 (5,9)	0,067	3 (11,5)		3 (6,1)	0,067
Não	18 (78,3)	59 (88,1)		44 (80,0)	32 (94,1)		23 (88,5)		46 (93,9)	
Cansaço										
Sim	4 (17,4)	12 (17,9)	0,955	13 (23,6)	3 (8,8)	0,077	5 (19,2)		7 (14,3)	0,533
Não	19 (82,6)	55 (82,1)		42 (76,4)	31 (91,2)		21 (80,8)		42 (85,7)	
Tosse ou engasgo										
Sim	2 (8,7)	8 (11,9)	0,669	9 (16,4)	1 (2,9)	0,051	3 (11,5)		3 (6,1)	0,086
Não	21 (91,3)	59 (88,1)		46 (83,6)	33 (97,1)		23 (88,5)		46 (93,9)	
Dificuldade materna para amamentar										
Sim	11 (47,8)	31 (46,3)	0,897	30 (54,5)	11 (32,4)	0,041*	10 (38,5)		24 (49,0)	0,584
Não	12 (52,2)	36 (53,7)		25 (45,5)	23 (67,6)		16 (61,5)		25 (51,0)	
Dor ou ferida na mama										
Sim	4 (17,4)	4 (6,0)	0,097	7 (12,7)	1 (3,0)	0,117	2 (7,7)		3 (6,1)	0,247
Não	19 (82,6)	63 (94,0)		48 (87,3)	33 (97,0)		24 (92,3)		46 (93,9)	
Pouca produção láctea										
Sim	19 (82,6)	16 (23,9)	0,518	11 (20)	8 (23,5)	0,693	3 (11,5)		16 (32,7)	0,032*
Não	4 (17,4)	51 (76,1)		44 (80)	26 (76,5)		23 (88,5)		33 (67,3)	
Escape extraoral durante AM										
Sim	9 (39,1)	28 (41,8)	0,823	25 (45,5)	12 (35,3)	0,345	10 (38,5)		20 (40,8)	0,874
Não	14 (60,9)	39 (58,2)		30 (54,5)	22 (64,7)		16 (61,5)		29 (59,2)	
Língua projetada para fora durante AM										
Sim	8 (34,8)	28 (41,8)	0,554	24 (43,6)	12 (35,3)	0,436	13 (50,0)		20 (40,8)	0,166
Não	15 (78,3)	39 (58,2)		31 (56,4)	22 (64,7)		13 (50,0)		29 (59,2)	
Chupeta (pesquisada ao sexto mês e com um ano)										
Sim	2 (8,7)	25 (37,3)	0,010*	13 (23,6)	13 (38,2)	0,141	0 (0,0)		15 (30,6)	<0,001*
Não	21 (91,3)	42 (62,7)		42 (76,4)	21 (61,8)		26 (100,0)		34 (69,4)	
Bico intermediário de silicone (pesquisada ao sexto mês)										
Sim	4 (17,4)	19 (28,4)	0,298	15 (27,3)	8 (23,5)	0,695	5 (19,2)		11 (22,4)	0,116
Não	19 (82,6)	48 (71,6)		40 (72,7)	26 (76,5)		21 (80,8)		38 (77,6)	

Teste Qui-quadrado de Pearson

* Valor de p significante ≤0,05

Legenda: AME = aleitamento materno exclusivo; AM = aleitamento materno; n = frequência absoluta; % = frequência relativa

Não houve associação entre a postura habitual de lábios e língua predominantemente adotada pelos lactentes na avaliação (até os 4 meses) e a situação da amamentação no sexto ou no décimo segundo mês (Tabela 4).

**Tabela 4:** Associação da postura habitual (de língua e de lábios) na primeira avaliação com o aleitamento materno exclusivo e complementado no sexto mês e com o aleitamento materno no décimo segundo mês

Variável	AME no sexto mês (n=33)			AM no sexto mês (n=33)			AM no primeiro ano (n=20)		
	Sim n (%)	Não n (%)	Valor de p	Sim n (%)	Não n (%)	Valor de p	Sim n (%)	Não n (%)	*Valor de p
Postura habitual de lábios									
Lábios abertos	2 (28,6)	17 (65,4)	0,072	14 (58,4)	5 (55,6)	0,968	6 (85,7)	8 (61,5)	0,193
Lábios semiabertos	3 (42,8)	8 (30,8)		8 (33,3)	3 (33,3)		1 (14,3)	1 (7,7)	
Lábios fechados	2 (28,6)	1 (3,8)		2 (8,3)	1 (11,1)		0 (0)	4 (30,8)	
Postura habitual de língua									
Dentro da cavidade oral	4 (57,1)	7 (26,9)	0,313	8 (33,3)	3 (33,3)	0,907	2 (28,6)	4 (30,8)	0,326
Entre os roletes gengivais	2 (28,6)	11 (42,3)		9 (37,5)	4 (44,5)		1 (14,3)	6 (46,2)	
Sobre o lábio inferior	1 (14,3)	8 (30,8)		7 (29,2)	2 (22,2)		4 (57,1)	3 (23)	

Teste Qui-quadrado de Pearson

* Valor de p significante ≤0,05

Legenda: AME = aleitamento materno exclusivo; AM = aleitamento materno; n = frequência absoluta; % = frequência relativa

## DISCUSSÃO

O objetivo do presente estudo foi pesquisar a duração do AME e do AM em lactentes com T21 e verificar a associação entre as condições clínicas e miofuncionais orofaciais com a presença de AME e de AM no sexto mês e no primeiro ano de vida. Esta pesquisa, assim como outras^(6,7,10,19)^, encontrou baixa duração do tempo de AM em bebês com T21, sendo de 69 dias (DP=84, mínimo de 0 e máximo de 365 dias) e do AME de 61,8 dias (DP=75,5, mínimo de 0 e máximo de 180 dias). Um estudo^(10)^, realizado no Chile, encontrou frequência de 46,6% de AME aos seis meses em lactentes com T21. Uma revisão da literatura apontou variação 31,6 a 55,4% de bebês com T21 que receberam AME e de 23,3 a 50% que nunca foram amamentados^(7)^; o que diverge dos dados encontrados no presente estudo, em que 25,6% dos lactentes encontravam-se em AME no sexto mês e 36,7% nunca foram amamentados. Divergências entre os estudos podem estar relacionadas às características da população, diferenças socioeconômicas e sociodemográficas, visto que foram conduzidos em diferentes países. No estudo realizado no Chile^(10)^, por exemplo, 75% das participantes tiveram licença-maternidade de seis meses ou mais, o que pode ter influenciado positivamente no tempo de AME.

Com relação à duração de aleitamento, em um estudo, conduzido na Itália, pesquisadores^(6)^ verificaram que lactentes com T21 foram amamentados por 54 dias, enquanto lactentes sem a síndrome foram amamentados por até 164 dias. Dessa forma, o achado do presente estudo vai ao encontro da literatura no que se refere ao período de aleitamento materno aquém do recomendado.

Na análise das variáveis clínicas o sexo masculino apresentou maior prevalência de AME ou AM no sexto mês. Não foram encontrados estudos com lactentes com T21 que realizassem essa investigação. Um estudo brasileiro, encontrou que lactentes do sexo masculino sem a síndrome foram amamentados por mais tempo, quando comparados com o sexo feminino^(20)^. Um estudo indiano também encontrou uma menor duração do AM em meninas^(21)^. Entretanto, outro estudo, realizado no Senegal, encontrou que a duração do AME foi mais curta em lactentes do sexo masculino quando comparados ao sexo feminino^(22)^. Não foram encontradas possíveis explicações para o achado, mas acredita-se que comparações entre os sexos quanto à curva de crescimento e número de internações poderiam fornecer informações adicionais sobre o motivo de os meninos terem sido amamentados por mais tempo.

Houve associação entre estar em AM no décimo segundo mês de vida e apresentar doença pulmonar. Este achado é contrário ao que apresenta a literatura^(23)^. Uma revisão sistemática traz informações sobre as práticas de não amamentação representarem risco significativo para infecção respiratória aguda e hospitalização; enquanto a amamentação exclusiva por quatro/seis meses mostrou reduzir significativamente a hospitalização, o tempo de internação, a necessidade de oxigênio suplementar e de internação em unidades intensivas^(23)^. Acredita-se que outras variáveis não investigadas nesta pesquisa, atreladas à presença das doenças pulmonares, possam ter sido fatores de confusão (características craniofaciais, hipotonia dos músculos respiratórios, postura da criança, tipo de complicações pulmonares e entre outros fatores). Além disso, as doenças pulmonares constituem uma categoria ampla de condições clínicas, que incluem asma, bronquite, bronquiolite, pneumonias, entre outras, as quais não foram diferenciadas nesta pesquisa. Portanto, a relação entre AM e doenças pulmonares em lactentes com T21 precisa ser melhor investigada.

Foi possível observar que o uso de fórmula láctea se associou a não estar em AME ou AM no sexto e décimo segundo mês de vida. Esse achado concorda com a literatura^(24,25)^. Ao oferecer a fórmula láctea ao invés do seio materno, tem-se menor estímulo de sucção no peito, que leva à diminuição da produção láctea e culmina em desmame precoce^(24)^. Além disso, a fórmula é ofertada em mamadeira, que exige menos esforço para a sucção, sendo, muitas vezes, difícil cessar o uso desse utensílio para retorno da amamentação, uma vez que o lactente se acostuma com ele^(25)^. Estudo^(24)^ aponta que o uso de bicos artificiais pode ocasionar espaçamento das mamadas, trazendo impacto na produção de leite, acarretando confusão de bicos e fluxo e, consequentemente, desmame precoce.

Os resultados sugerem que amamentar na primeira hora de vida é importante para manutenção do AME e AM até o sexto mês, bem como do AM até o décimo segundo mês, corroborando pesquisas anteriores que apontam que o contato precoce do recém-nascido com o seio materno na primeira hora de vida exerce influência no início, manutenção e duração do AME^(26)^. Além disso, o contato pele a pele na primeira hora de vida favorece o sucesso da amamentação, inclusive da amamentação exclusiva por maior tempo^(26)^. Apesar disso, um estudo, realizado na Suécia, apontou, pelo relato de mães de bebês com T21, que a amamentação durante a primeira hora de vida não foi encorajada pelos profissionais de saúde, independente da situação clínica desses bebês^(8)^. O fato de não ter sido investigado, na presente pesquisa, se a ausência da amamentação na primeira hora se deu pelas condições clínicas do bebê ou não, constituiu uma limitação do estudo. Sabe-se que a T21 pode vir associada a várias comorbidades^(4)^, que não foram investigadas na presente pesquisa, e que podem dificultar a amamentação^(9)^, até mesmo a quantidade de internações, de cirurgias e outros procedimentos hospitalares que separam a díade. Um estudo realizado com mães de bebês com T21, em Porto Rico, verificou que 50% delas não puderam ficar junto de seus bebês durante a permanência no hospital^(11)^.

Já na análise das variáveis referentes ao relato da mãe, houve associação entre percepção materna de dificuldade do lactente para ser amamentado com presença de AM no sexto mês. Tal associação pode ser justificada pelo fato de as mães que relataram essa dificuldade, estarem amamentando. Dessa forma, perceberam as reais dificuldades do lactente em questão. Tal percepção esteve atrelada aos seis primeiros meses, possivelmente pelo fato de os primeiros meses serem os mais difíceis nesse processo^(27)^. A literatura aponta a relação entre primiparidade e dificuldade materna durante a amamentação^(28)^, entretanto na presente pesquisa não coletamos essa variável em questão. Dentre as dificuldades pesquisadas, parece ser a pega a principal questão, dada a associação também encontrada entre estar em AM no sexto mês e apresentar dificuldade relacionada à pega. A dificuldade com a pega na T21 é justificada, na literatura, pela hipotonia dos músculos orbicular da boca e bucinador, característica da T21^(1)^, que compromete a geração da pressão negativa intraoral, importante para a extração do leite^(5,13)^. Ademais, no processo de extração do leite em seio materno, há grande participação dos músculos elevadores da mandíbula, os quais também podem estar hipotônicos em lactentes com a síndrome^(5)^.

Mães que amamentaram de forma complementada até o sexto mês de vida seus bebês, relataram, em maior proporção, dificuldades de origem materna durante a amamentação, sendo elas dor/ferida na mama e/ou diminuição do leite, o que, novamente, pode ser justificado pelo fato de estarem amamentando e perceberem tais dificuldades. Nesse contexto, houve associação entre baixa produção láctea e ausência de AM no décimo segundo mês, o que ocorre pela falta de estímulo de sucção do bebê ao seio materno, diminuindo, por consequência, a produção de leite^(24)^.

O uso de chupeta impactou diretamente em não estar em AME no sexto mês ou em AM no décimo segundo mês. Esse achado pode ser explicado pelo fato de que o uso da chupeta reduz a frequência da amamentação, comprometendo, assim, a produção de leite materno e, consequentemente, oferecendo risco à interrupção da amamentação. Um estudo epidemiológico, que comparou o uso de chupeta e a duração do AM, comprovou que lactentes que não usaram o utensílio apresentam quatro vezes maior probabilidade de continuar a amamentação até o sexto mês em comparação com os que fizeram o uso em tempo integral^(29)^. Já em 2017, um estudo observacional identificou associação entre o uso da chupeta e a interrupção do AME, concluindo que o uso da chupeta é fator de risco para interrupção do AME em lactentes menores de seis meses^(30)^. Ademais, em uma metanálise, identificou-se que o uso da chupeta aumentou em duas vezes o risco de interrupção do AM, além disso referiu-se que a chupeta também é considerada um marcador de dificuldades relacionadas à amamentação, bem como a diminuição da motivação materna para amamentar^(31)^.

Esperava-se encontrar associação entre a postura habitual de lábios e língua e a presença do AME e AM. A associação pode não ter sido encontrada devido ao tamanho da amostra para análise desses e o tempo de duração dos vídeos, de apenas cinco minutos, que pode não refletir a postura de fato adotada na maior parte do tempo pelos lactentes, retratando apenas um momento específico. Entretanto, apesar de não ter apresentado significância estatística, notou-se que lactentes com postura habitual de lábios fechados nos primeiros quatro meses, encontravam-se mais comumente em AME ao sexto mês, ao contrário daqueles que apresentavam lábios abertos, podendo tal achado ser justificado pela hipotonia do músculo orbicular da boca, presente nos casos de postura de lábios abertos, que impacta diretamente na pega em seio materno, influenciando na duração e manutenção do AME até o sexto mês de vida^(2)^. Nesse mesmo raciocínio, dentre os lactentes amamentados de forma exclusiva ou não ao sexto mês, encontra-se maior proporção de língua posicionada dentro da cavidade oral sendo considerada dentro da cavidade oral a língua plana com ponta arredondada (repouso no assoalho da boca), ou elevada (repouso no palato), bem como quando não era visível (lábios fechados), as quais são posturas relacionadas a um melhor tônus da musculatura da língua em comparação com a língua que adota uma postura habitual entre os roletes gengivais ou sobre o lábio inferior. A literatura aponta que lactentes sem alteração no frênulo lingual tendem a manter a postura habitual da língua elevada^(32)^. Dessa forma, a língua elevada, acoplada ao palato, tem sido considerada o padrão de normalidade^(32,33)^. Porém, o foco desta pesquisa, no que se refere à posição da língua, foi direcionado apenas para a sua relação anteroposterior, ou seja, o quão anteriorizada a língua se encontrava dentro da cavidade oral, visto que a protrusão lingual é característica geralmente reportada em indivíduos com T21.

O estudo em questão comprovou a hipótese de que a duração do AM apresenta associação com uso de fórmula, dificuldades maternas e dos lactentes e uso de chupeta. Ademais, a partir dos resultados obtidos, pode-se ter melhor compreensão dos fatores que influenciam na amamentação de lactentes com a T21, visto que na literatura ainda existe uma escassez de estudos nessa população em específico. Os impactos das comorbidades e hábitos orais desses lactentes interferem, diretamente, no desmame precoce e, por consequência, podem influenciar no desenvolvimento das estruturas orofaciais, além das funções de mastigação, sucção e deglutição; sendo que o sucesso no desenvolvimento das funções garante melhora no tônus dessas estruturas.

Consideram-se limitações do estudo o número reduzido de participantes, ausência de avaliação do modo respiratório, o fato de as informações terem sido baseadas na aplicação de questionários que levaram em conta a percepção materna sobre os aspectos pesquisados. Entretanto, considerar a percepção materna é importante no processo de compreender os principais fatores que influenciam na duração e manutenção do AME e AM. A subjetividade relacionada à análise da postura habitual de lábios e de língua também pode ser citada como limitação. No intuito de minimizar a subjetividade inerente à coleta de dados, para avaliação da postura habitual realizou-se uma análise quantitativa, baseada em vídeos, com verificação da concordância intra e interavaliador, o qual pode ser considerado um ponto forte da pesquisa. O fato de a comparação intra-avaliador ter sido classificada como excelente e a comparação interavaliador como substancial demonstra que houve boa reprodutibilidade na análise dos vídeos, o que aumenta a confiabilidade dessas análises. Os achados desta pesquisa podem ser úteis para que os profissionais da saúde possam orientar da melhor forma os pais e/ou responsáveis por lactentes com T21 em prol na manutenção do aleitamento nessa população.

## CONCLUSÃO

Este estudo encontrou baixa prevalência de AME no sexto mês e de AM no sexto e no décimo segundo mês em lactentes com T21.

Houve associação entre sexo masculino do lactente e presença de AME e de AM no sexto mês; entre ter amamentado na primeira hora de vida e presença de AME no sexto mês e de AM no sexto e no décimo segundo mês; entre fazer uso de fórmula e ausência AME no sexto mês e de AM no sexto e no décimo segundo mês; entre presença de doença pulmonar e de AM no décimo segundo mês; e entre uso de chupeta e ausência de AME no sexto mês e de AM no décimo segundo mês.

Mães que estavam em AM no sexto mês relataram com mais frequência dificuldade do lactente e delas próprias para amamentar, com associação entre relato de dificuldade na pega e presença de AM no sexto mês e relato de baixa produção láctea e ausência de AM no décimo segundo mês.
